# Distinct Pharmacological Properties of Gaseous CO and CO-Releasing Molecule in Human Platelets

**DOI:** 10.3390/ijms22073584

**Published:** 2021-03-30

**Authors:** Patrycja Kaczara, Kamil Przyborowski, Tasnim Mohaissen, Stefan Chlopicki

**Affiliations:** 1 Jagiellonian Centre for Experimental Therapeutics, Jagiellonian University, (JCET), Bobrzynskiego 14, 30-348 Krakow, Poland; kamil.przyborowski@jcet.eu (K.P.); tasnim.mohaissen@jcet.eu (T.M.); stefan.chlopicki@jcet.eu (S.C.); 2 Faculty of Pharmacy, Jagiellonian University Medical College, Medyczna 9, 30-688 Krakow, Poland; 3 Faculty of Medicine, Chair of Pharmacology, Jagiellonian University Medical College, Grzegorzecka 16, 31-531 Krakow, Poland

**Keywords:** platelet aggregation, carbon monoxide, soluble guanylate cyclase, energy metabolism, CO-RMs

## Abstract

Carbon monoxide (CO)—gaseous or released by CO-RMs—both possess antiplatelet properties; however, it remains uncertain whether the mechanisms involved are the same. Here, we characterise the involvement of soluble guanylate cyclase (sGC) in the effects of CO—delivered by gaseous CO–saturated buffer (CO_G_) and generated by CORM-A1—on platelet aggregation and energy metabolism, as well as on vasodilatation in aorta, using light transmission aggregometry, Seahorse XFe technique, and wire myography, respectively. ODQ completely prevented the inhibitory effect of CO_G_ on platelet aggregation, but did not modify antiplatelet effect of CORM-A1. In turn, CO_G_ did not affect, whereas CORM-A1 substantially inhibited energy metabolism in platelets. Even though activation of sGC by BAY 41-2272 or BAY 58-2667 inhibited significantly platelet aggregation, their effects on energy metabolism in platelets were absent or weak and could not contribute to antiplatelet effects of sGC activation. In contrast, vasodilatation of murine aortic rings, induced either by CO_G_ or CORM-A1, was dependent on sGC. We conclude that the source (CO_G_ vs. CORM-A1) and kinetics (rapid vs. slow) of CO delivery represent key determinants of the mechanism of antiplatelet action of CO, involving either impairment of energy metabolism or activation of sGG.

## 1. Introduction

Carbon monoxide (CO) produced endogenously by heme oxygenase (HO) enzymes exerts antithrombotic action [1,2,3]. Exogenous CO, delivered by inhalation with CO gas [1] or in the form of CORM-2 [3] to HO-1–deficient animals, inhibited thrombosis. Numerous mechanisms may be involved in the antithrombotic action of CO, including its antiplatelet activity. Indeed, antiplatelet effects resulting from the activation of HO-1/CO pathway were confirmed in several studies. For example, induction of HO-1 in vivo reduced platelet adhesion to sinusoidal endothelial cells (ECs) [4]; aggregation of platelets co-incubated in vitro with ECs overexpressing HO-1 or with downregulated HO-1 was reduced or enhanced, respectively [5]. In turn, a transfer of HO-1–expressing wild-type platelets to HO-1–deficient aortic graft recipients prolonged animal survival due to reduced thrombosis [3]. Finally, CO–releasing molecules (CO-RMs, CORM-A1 and CORM-3) inhibited activation of platelets in vivo, supporting the notion that exogenously delivered CO displays an antiplatelet effect [6,7].

Generally, in the field of CO research, it is accepted that either form of CO delivery induces biological effects by the same mechanism, which is determined by local concentration of CO. Carbon monoxide—gaseous or released by CO-RMs—induces antiplatelet effects in in vitro models [6,8,9,10,11]. However, previous reports provided by independent groups claimed that the antiplatelet effect of CO was mediated by soluble guanylate cyclase (sGC) when platelets were treated with gaseous CO [8], or independent of sGC when platelets were treated with CO-RMs [9,10,11]. CO gas and CO-RMs are applied interchangeably; however, the mechanisms of antiplatelet effects induced by these two systems have not been compared directly as yet.

We demonstrated recently that CORM-A1 slowly releasing CO (t_1/2_ = 21 min [12]) induced antiplatelet effect by the inhibition of oxidative phosphorylation and depletion of NAD^+^, resulting in the inhibition of glycolysis; subsequently simultaneous inhibition of these processes led to ATP depletion [11]. In turn, in another type of tissue, namely murine aortic rings, a vasodilatation induced by CO was independently of the source of CO mediated by sGC activation, as reviewed by Foresti et al. [13]. To explain this puzzling discrepancy, we set out to compare in the same experimental settings the effects of CO delivered by gaseous CO (CO_G_) or released from CORM-A1 on platelet aggregation and energy metabolism, with the aim to delineate the involvement of sGC. For comparison, we re-analyzed the involvement of sGC in vasodilatation induced by CO_G_ and CORM-A1 in pre-constricted aortic rings.

## 2. Results

### 2.1. Effect of Carbon Monoxide on Platelet Aggregation—Involvement of sGC

**Figure 1:**
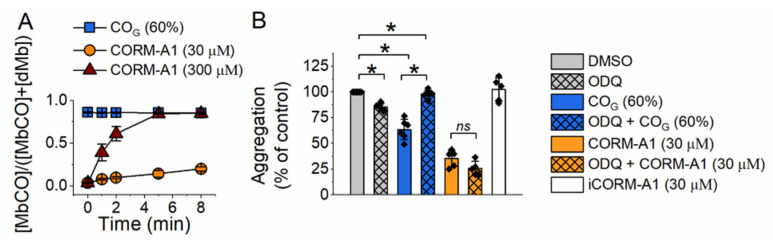
Comparison of the effects of CO delivered by CO-saturated buffer or released by CORM-A1 on platelet aggregation. (**A**) Time and concentration–dependent changes in fraction of carboxy-Myoglobin (MbCO), after mixing of CO-saturated buffer (CO_G_; 60%) or CORM-A1 with deoxy-Myoglobin (dMb, 66 µM). (**B**) Effects of ODQ (10 µM) on collagen (2 µg/mL)–induced aggregation of WP treated with CO_G_ (60%) or CORM-A1 (30 µM); effect of inactive CORM-A1 (iCORM-A1; 30 µM). Data represent means +/-SD from at least three independent experiments. * *p* < 0.05.

Using a myoglobin assay we confirmed that the concentration of CO delivered by CORM-A1 (30 µM) after 8 min of incubation was significantly lower than the concentration of CO delivered by gaseous CO–saturated buffer (CO_G_; 60% *v*/*v*; Figure 1A). Importantly, the dynamics of CO availability for these two approaches of CO delivery were substantially different—rapid for CO_G_ and slow for CORM-A1. Of note, even though CORM-A1 delivered much less CO to platelets, it induced stronger inhibition of platelet aggregation induced by collagen (2 µg/mL) (Figure 1B). CO_G_ (60% *v*/*v*, which is equivalent to around 0.6 mM concentration of CO) reduced platelet aggregation to 63 +/− 9.9%, whereas CORM-A1 (30 µM) to 35 +/− 8.2% of control (300 µM CORM-A1 completely inhibited platelet aggregation; data not shown). Inactive CORM-A1 (iCORM-A1, 30 µM) did not affect platelet aggregation (Figure 1B), supporting that the effect of CORM-A1 was indeed related to CO release. Interestingly, ODQ (10 µM, 1 min of pre-incubation with platelets) abrogated the effect of CO_G_, but did not modify inhibition of platelet aggregation induced by CORM-A1 (Figure 1B). These data confirm that CO inhibits platelet aggregation by distinct mechanisms, depending on whether it is delivered as CO_G_ or CORM-A1.

### 2.2. Effect of Carbon Monoxide on Platelet Energy Metabolism

**Figure 2:**
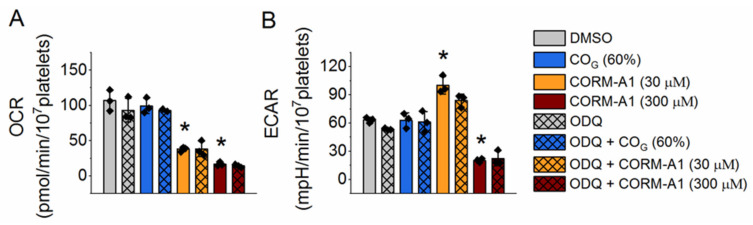
Distinct effects of CORM-A1 and CO gas on platelet energy metabolism. (**A**) Oxygen consumption rate (OCR) and (**B**) extracellular acidification rate (ECAR) were analyzed by Seahorse XF96 Analyser. Platelets were treated with DMSO or ODQ or CO_G_ (60%) or CORM-A1 (30 or 300 µM) just before the start of the Seahorse assay; the readouts were taken 30 min after treatment. Data represent means +/-SD from at least three independent experiments. * *p* < 0.05 as compared with the corresponding control.

We demonstrated previously that CORM-A1 inhibited platelet aggregation through inhibition of platelet energy metabolism [11]. To compare directly the effects of CO_G_ and CORM-A1 on platelet energy metabolism we measured oxygen consumption rate (OCR; reflecting mitochondrial respiration) and extracellular acidification rate (ECAR; reflecting glycolysis). Platelets were treated with tested compounds just before starting the Seahorse assay, so the readings presented in Figure 2 were taken 30 min after the start of treatment. CO_G_ (60%) did not affect OCR or ECAR, independently of the presence or absence of ODQ (10 µM). In contrast, as demonstrated in Figure 2, CORM-A1 (30, 300 µM) decreased OCR at both applied concentrations. However, ECAR increased in response to 30 µM and decreased in response to 300 µM CORM-A1. ODQ did not modify the CORM-A1 effects on energy metabolism.

### 2.3. Effect of sGC Activation on Platelet Aggrgation and Energy Metabolism

**Figure 3:**
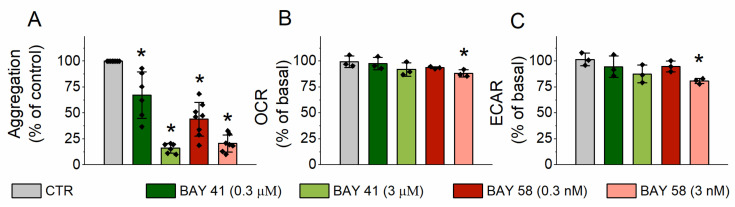
The effects of soluble guanylate cyclase (sGC) activation on platelet aggregation and energy metabolism. Platelets treated with DMSO (as a vehicle; 0.1%), BAY 41-2272 (0.3 or 3 µM) or BAY 58-2667 (0.3 or 3 nM). (**A**) Aggregation of platelets activated with collagen (2 ug/mL), (**B**) changes in oxygen consumption rate (OCR; 30 min after addition of tested compounds) presented as % of basal OCR and (**C**) changes in oxygen consumption rate (OCR; 30 min after addition of tested compounds) presented as % of basal. Data represent means +/-SD from et least three independent experiments. * *p* < 0.05 as compared with the CTR group.

To further confirm the distinct mechanisms of antiplatelet effects of CO_G_ and CORM-A1 we tested whether the direct activation of sGC could modulate energy metabolism in platelets. Even though BAY 41-2272 (sGC stimulator) or BAY 58-2667 (sGC activator) significantly inhibited platelet aggregation (Figure 3A) their effects on energy metabolism, specifically mitochondrial respiration (Figure 3B) and glycolysis (Figure 3C), were absent or weak and could not contribute to antiplatelet effects induced by sGC activation.

### 2.4. Effect of Carbon Monoxide on Aortic Rings Vasodilatation

**Figure 4:**
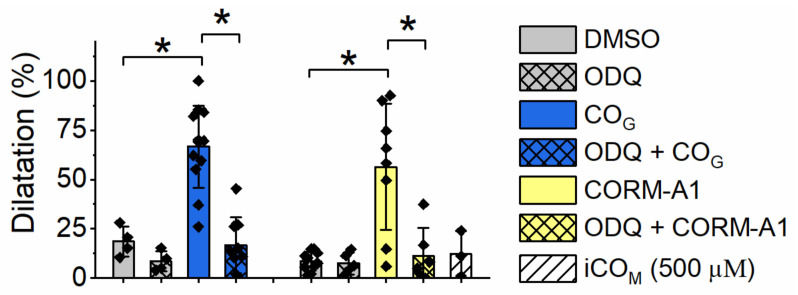
Comparison of the effects of CO delivered by CO-saturated buffer or released by CORM-A1 on vasodilatation in aortic rings. Aortic rings dilatation was evaluated 5 min after addition of CO–saturated assay buffer (CO_G_; 60%) or 30 min after addition of CORM-A1 (500 µM) or iCORM-A1 (500 µM). Data represent means +/-SD from at least three independent experiments. * *p* < 0.05.

CO induced vasodilatation of isolated vascular preparations of the murine aorta in both conditions, when delivered by CO_G_ (60%) as well as when released from CORM-A1 (500 µM) (Figure 4). In either case the effect was dependent on activation of sGC, as ODQ abrogated the CO–induced vasodilatation. Importantly, the dynamics of CO availability for these two ways of CO delivery were substantially different, and because of these the vasodilatation was measured after 5 min of exposition to CO_G_ and after 30 min of exposition to CORM-A1. However, eventually CO_G_– and CORM-A1–delivered CO resulted in a similar degree of vasodilatation.

## 3. Discussion

It is generally accepted that CO acts through the same mechanism in the same experimental system, irrespectively whether delivered as gaseous CO (CO_G_) or released from CO–releasing molecules (CO-RMs). However, previous reports from independent groups claimed that antiplatelet effect of CO was [8,14] or was not [9,10,11] mediated by soluble guanylate cyclase (sGC). Remarkably, such discrepancy has not been explained until now. In the present work, to the best of our knowledge for the first time, we directly compare the mechanism of antiplatelet effects of CO delivered by gaseous CO–saturated buffer or generated by CORM-A1 (a molecule slowly releasing CO [12]). Our results clearly showed that inhibition of platelets by CO_G_ and CORM-A1—even though similar in magnitude—involved distinct mechanisms. Antiplatelet effects of CO_G_ and CORM-A1 were differentially affected by ODQ, suggesting that the mechanisms of action were dependent and not on sGC, respectively (Figure 1B). The most intriguing observation was that CO liberated from 30 μM CORM-A1, which after 8 min achieved much lower concentration than when delivered by CO–saturated buffer (CO_G_) (Figure 1A), displayed a stronger effect on platelet aggregation (Figure 1B).

We demonstrated recently that CO generated by CORM-A1 inhibited platelet aggregation through inhibition of energy metabolism and depletion of NAD^+^ [11]. Brüne and Ullrich showed that gaseous CO inhibited platelets’ aggregation by activation of sGC [8], which we have confirmed in the present work using a gaseous CO–saturated buffer (CO_G_; Figure 1B). The effect of CO was reversed by Brüne and Ullrich after irradiation of platelets with visible light leading to dissociation of CO [8]. Moreover, the inhibition of platelet aggregation by gaseous CO was lost in the presence of a sGC inhibitor—ODQ. All these data suggested the binding of CO to the ferrous heme regulatory subunit of sGC, activation of sGC and subsequent antiplatelet effect. In fact, although sGC is the major target for nitric oxide (NO) [15], it could be also sensitive to CO. However, the responsiveness of sGC to NO and CO is quite different, as purified sGC was activated only 4.4-fold by nearly 100% CO as compared with 130-fold activation by 0.5% NO [16]. Furthermore, NO and CO bind to sGC differently and cause conformational changes of the enzyme, resulting in different levels of its activation [17,18]. NO, through a transient 6-coordinated NO–bond state followed by heme-His bond break, forms an activated 5-coordinated NO–bond state of sGC [17,19], whereas CO forms 6-coordinated CO–bond state of sGC, which is much less active than NO–bond sGC [16,17]. The activation of sGC by CO, as well as activation by NO, were both shown to be highly enhanced by YC-1 [16,17,20] or other compounds sensitizing sGC, for example BAY 41-2272, riociguat, nelociguat, vericiguat [18]. These compounds provoke conformational changes of sGC facilitating heme-His bond break, leading to formation of 5-coordinated CO–bond state, and increasing sGC activity, similarly to NO–bond sGC. The important finding of the present report was that both stimulator (BAY 41-2272) and activator (BAY 58-2667) of sGC even though significantly inhibited platelet aggregation had a very weak effect on platelet energy metabolism, providing evidence that alterations in energy metabolism could not contribute to antiplatelet effects of sGC activation (Figure 3). Thus, the CO–induced sGC–dependent inhibition of platelet aggregation was independent of energy metabolism in platelets.

In the present work we also showed that even though CO_G_ significantly reduced platelet aggregation (Figure 1B), it did not affect energy metabolism (Figure 2). On the other hand, CORM-A1 significantly reduced mitochondrial respiration (reflected by OCR)—at 30 μM concentration to the level almost as low as at 300 μM concentration. Since CO binds efficiently to cytochrome c oxidase [21,22], it was unexpected to see the lack of CO_G_ effects on platelet mitochondrial respiration, after all, CO_G_ delivered more CO than 30 μM CORM-A1 (Figure 1A). D’Amico and collaborators treated HEK293 cells with 20 μM CO delivered by gaseous CO–saturated water and demonstrated inhibition of mitochondrial respiration to around 65% of basal, however the experiments were performed in a gas-tight chamber [23]. In contrast, our experimental system was open; not only the Seahorse XFe multiwall plate used for measurements of oxygen consumption but also the cuvette used for measurements of platelet aggregation were both open, and involved mixing of the assay medium or buffer at 37 °C, what might facilitate escaping of CO. However, binding of CO to cytochrome c oxidase is expected to be irreversible—we demonstrated previously that inhibition of mitochondrial respiration in breast cancer cells was gradually progressing after addition of CO donor (CORM-401), in contrast to the effect of NO (delivered by PAPA NONOate), which was being gradually reversed during escaping of NO [24]. Thus, we expected a rapid and permanent binding of CO_G_ to cytochrome c oxidase and inhibition of mitochondrial respiration. Mixing of CO–saturated assay medium (around 1 mM CO) with CO-free assay medium in a ratio of 60:40 allows to obtain nearly 600 µM CO at room temperature as a bolus [refer to Materials and Methods section]. CO at such high concentration is expected to bind irreversibly to cytochrome c oxidase and to inhibit this enzyme. A possible explanation of the lack of mitochondrial respiration inhibition under CO_G_ is that CO at high concentrations and with ambient oxygen level might have a higher affinity to sGC and/or other heme-containing proteins than to cytochrome c oxidase.

In our previous work we showed that CO delivered by CORM-A1 modulated glycolytic flux in a biphasic manner—activated for lower and reduced for higher concentrations of CORM-A1 [11]. The data presented in Figure 2B confirm this effect also in experimental conditions applied in the present work. An increase of glycolysis for lower concentrations of CORM-A1 was a compensatory response to inhibition of mitochondrial respiration; however, a decrease of glycolysis for higher concentrations of CORM-A1 resulted from NAD^+^ depletion, which is necessary for GAPDH activity [11]. Interestingly, exogenous pyruvate prevented the inhibition of platelet aggregation induced by CORM-A1, but also normalized platelet energy metabolism (Supplementary Figure S1), and this effect was most likely due to increased cytosolic NAD^+^ availability [11]. CO_G_ did not affect platelet energy metabolism, and pyruvate did not modify CO_G_–induced inhibition of platelet aggregation nor energy metabolism (Supplementary Figure S1). The data further confirmed the distinct pharmacological properties of CO_G_– and CORM-A1–derived CO showing their distinct dependence on NAD^+^ availability.

It is commonly believed that the effects of CO-RMs result not only from activity of released CO, but also from the rest of molecule. To confirm that the effect of CORM-A1 indeed resulted from CO we performed experiments with inactive CORM-A1 (iCORM-A1), which did not affect the analyzed parameters. Most of available CO-RMs contain transition metal ions, but water-soluble CORM-A1 slowly liberating CO (t_1/2_ = 21 min) is made of carboxylic acid, which contains non-metal boron [12]. CORM-A1, by delivery of controlled low amounts of CO, with slow kinetics may thus became a therapeutically useful compound. Furthermore, other CO-RMs slowly liberating CO had the ability to inhibit platelet aggregation in vitro comparable to CORM-A1 [25], in contrast to CORM-3 (t_1/2_ = 1 min), which was less efficient than CORM-A1 [10].

It is not clear whether CORM-A1 or other available CO-RMs may cross biological membranes and enter intracellular compartments. It is not yet possible to precisely measure CO produced endogenously in platelets or platelets’ environment, but it can be expected that the kinetics of CO availability derived from HO-1 is distinct as compared with delivery by gaseous CO–saturated buffer (CO_G_) or CORM-3, which quickly releases CO. Peng and collaborators demonstrated that hemin–induced production of cGMP in platelets was not attenuated in mice lacking HO-1, as compared with wild-type animals, leading to the conclusion that the action of HO-1–derived CO was not mediated by activation of sGC [26]. Endogenous production of CO can be mimicked by CO-RMs enabling delivery of controlled amounts of CO relatively slowly, for example CORM-A1, which, endowed with a promising pharmacological profile to inhibit platelet aggregation [6,10,11], represent a prototypic CO-RM–based antiplatelet agent.

Based on the results presented here we conclude that the mechanism of action of CO liberated at low concentrations from CORM-A1 is independent of sGC activation, contrary to the mechanism of action of CO delivered at high, non-physiological concentrations. In this context, CO-RMs slowly releasing CO are emerging as a new strategy for antiplatelet therapies.

Interestingly, in contrast to platelets in aortic rings, vasodilatation induced by CO was mediated by sGC, irrespective of the source of CO, as evidenced by the use of ODQ (Figure 4). Even though the dynamics of CO availability for these two ways of CO delivery was different, they finally reached similar local concentrations and induced similar degrees of vasodilatation. A previous study has described the role of sGC in CO–induced vasodilatation repeatedly [13]. It was demonstrated that CO delivered by CORM-3 stimulated vasodilatation via increased NO production, rather by a direct activation of sGC [27]. Some reports also confirm [27,28,29,30] and the others exclude [31] the involvement of large-conductance calcium-regulated potassium ion channels (BKca) in CO–dependent vasodilatation. Failli and collaborators proved that vasodilatation dependent on NO and CO was predominantly mediated by, respectively, sGC and potassium channels [30]. Nevertheless, the pathways activated by NO and CO are not separated - they interact and enhance each other. Altogether, we confirmed that the vasodilatory action of CO delivered by CORM-A1 or CO_G_ was in either case mediated via activation of sGC. CO–induced vasodilatation could of course also involve other previously postulated mechanisms, such as an NO–dependent pathway, and other co-existing mechanisms stimulating sGC or sensitizing sGC to CO. It may be of note that we did not dismiss the possibility that CO–mediated vasodilatation in the aorta involved alterations in energy metabolism. Interestingly, hydrogen sulfide induced vasorelaxation via metabolic inhibition independently of sGC [32], but the inhibition of energy metabolism pathways impaired the relaxation of aortic rings [33,34,35].

The differences in responses of various tissues to CO, as demonstrated here for platelets and vascular wall may depend on the concentrations and distributions of various heme-containing proteins, which can compete for CO binding. Several cellular targets for CO have been identified and described, as reviewed by Foresti et al. [13]. The complexity of biological systems makes it difficult to precisely distinguish the effects of CO on all the possible targets and further studies would be needed. Despite these limitations, CO induced clearly mechanistically different effects when delivered to platelets, rapidly or slowly, at high or low concentration, by CO_G_ or CORM-A1, respectively.

In summary, even though the final local concentrations of CO delivered by gaseous CO–saturated buffer or CORM might reach eventually similar levels in the same experimental system, the source (CO_G_ or CORM-A1) and kinetics (rapid or slow) of CO delivery seem to be the key determinants of the mechanism of antiplatelet CO action, involving either the impairment of energy metabolism or the activation of sGC without effects on energy metabolism. Quite surprisingly, the mechanism of CORM-A1–induced antiplatelet effect was not shared by CO_G_, which activated distinct intraplatelet pathways. In contrast, the mechanism of the vasodilatory action of CORM-A1 and CO_G_ was the same. New paradigms are needed in the CO pharmacology to accommodate these findings.

## 4. Materials and Methods

### 4.1. Materials

Dulbecco’s Phosphate Buffered Saline without Ca^2+^ and Mg^2+^ (DPBS; Lonza); Seahorse XF Base Medium Minimal DMEM (Agilent Technologies); acetylcholine, CORM-A1, glucose, glutamine, phenylephrine, SNP, sodium hydroxide, sodium pyruvate (Sigma-Aldrich); collagen (CHRONO-LOG); bovine serum albumin (SERVA); dimethyl sulfoxide (DMSO; Life Technologies); gaseous carbon monoxide (Multax). Inactive CORM-A1 (iCORM-A1) was prepared according to the protocol described by Motterlini et al. [12]. For the experiments with gaseous CO, an assay buffer was saturated with CO gas by bubbling for 30 min at room temperature (as described by Rotko et al. [36]) and used immediately. As solubility of CO in water is 2.3 mL/100 mL at 20 °C [37], saturation of a water–based assay buffer with pure CO gas allows to achieve at room temperature around 1 mM solution of CO.

### 4.2. Animals

C57/Bl6 mice were anesthetized intraperitoneally with a mixture of ketamine and xylazine in doses of 100 and 10 mg/kg b.w., respectively. A thoracic aorta was cleaned from any adherent tissues and cut into 2 mm–long pieces; the aortic rings were placed in cold Krebs-Henseleit solution (KH) (NaCl 118.0 mM, CaCl_2_ 2.52 mM, MgSO_4_ 1.16 mM, NaHCO_3_ 24.88 mM, K_2_PO_4_ 1.18 mM, KCl 4.7 mM, glucose 10.0 mM; pH = 7.4).

### 4.3. Isolation of Human Platelets

Venous blood was obtained from male volunteers at the University Hospital Blood Bank Centre. Volunteer donors had not taken any medicines for the preceding two weeks. Informed consent was given by a volunteer prior to the blood withdrawal and study conformed to the principles outlined in the World Medical Association (WMA) Declaration of Helsinki. Blood obtained from at least three donors per one independent experiment was collected into tubes containing sodium citrate (3.2%, 9:1 *v*/*v*) as an anti-coagulant agent. To obtain platelet-rich plasma (PRP), blood was centrifuged (260g, 15 min). The platelet pure plasma fraction (blank sample) was obtained by centrifugation of the remaining blood (2600 g, 20 min). Washed platelets (WP) were obtained from PRP, by a centrifugation/washing cycle using prostacyclin (PGI_2_)-containing phosphate buffered saline (PBS containing albumin (1 g/L) and glucose (1g/L)), according to the previously described method [11]. WP were finally suspended in assay buffer (PBS supplemented with glucose (1 g/L) and glutamine (2 mM), pH 7.4; for platelet aggregation analysis) or assay medium (Seahorse XF Base Medium Minimal DMEM supplemented with glucose (1 g/L) and glutamine (2 mM), pH 7.4; for measurements of oxygen consumption rate and extracellular acidification rate) at a density of 200,000 platelets/µL, unless otherwise stated. Contamination of neutrophils in WP was less than 1/10^6^ platelets.

### 4.4. Platelet Aggregation Assay

Aggregation of washed human platelets (WP) was assessed using a dual channel aggregometer (CHRONO-LOG), as described previously [11]. Briefly, 500 µL of WP (at a density of 200,000/µL) untreated or treated with tested compounds were equilibrated for 2 min at 37 °C with a continuous stirring (800 rpm) and then stimulated with collagen (2 µg/mL) to cause aggregation followed by transmittance measurements for 6 min. For experiments with CORM-A1, platelets were suspended at a density of 200,000 platelets/µL and 5 µL of CORM-A1 was added to 500 µL of platelet suspension. For experiments with gaseous CO, platelets were suspended at a density of 500,000 platelets/µL and 300 µL of CO-saturated assay buffer was added to 200 µL of platelet suspension (final density of platelets was 200,000/µL with 60% of CO–saturated buffer).

### 4.5. Analysis of Cellular Bioenergetics Using Seahorse Extracellular Flux Technology

To measure mitochondrial function and glycolysis in isolated washed human platelets, a Seahorse XFe96 Analyzer was used as described previously [11]. Briefly, freshly isolated platelets were suspended in assay medium (bicarbonate-free low buffered DMEM supplemented with glucose (1 g/L) and glutamine (2 mM), pH 7.4), and introduced into the Seahorse XFe96-well plate (10 × 10^6^ platelets in 80 µL per well), followed by centrifugation (700 g, 5 min) and incubation in air without CO_2_ (1h, 37 °C). Just before the start of measurement, either a control assay medium, an assay medium saturated with CO or an assay medium containing CORM-A1 were added to wells in volumes of 120 µL. Followed by 12 min of equilibration, oxygen consumption rate (OCR) and extracellular acidification rate (ECAR) were assessed over time. In experiments on platelets treated with BAYs, the compounds were added from the ports after three initial measurements. The data presented in the figures were taken 30 min after addition of tested compounds.

### 4.6. Myoglobin Assay for Quantification of CO

For quantification of CO we used a well-validated method of myoglobin assay. The assay was performed as described by Fayad-Kobeissi [38] with minor changes. Heart myoglobin was dissolved in phosphate-buffered saline (PBS, pH 7.4) at a concentration of 100 µM, converted to deoxymyoglobin (dMb) with sodium dithionite (0.2%) and maintained at 37 °C. PBS saturated with gaseous CO was mixed with fresh PBS giving 60% of CO–saturated buffer; CORM-A1 was added to PBS to produce 30 or 300 µM concentrations; as a control PBS without CO was used. PBS (control) or PBS containing CO (gaseous or delivered by CORM-A1) were mixed with dMB in a ratio of 1:2 (vol:vol), incubated at 37 °C and taken after 1, 2, 5 or 8 min of incubation for recordings of dMb (without CO) and carboxymyoglobin (MbCO) spectra (500–600 nm) using UV-VIS spectrometer (Perkin Elmer) at room temperature. A fraction of carboxymyoglobin [MbCO] of total myoglobin ([MbCO]+[dMb]) was calculated as described by Hasegawa et al. [39].

### 4.7. Assessment of Endothelial Function in Isolated Mouse Aortic Rings

The assay was performed as described previously [40,41]. Briefly, aortic rings were mounted in a Mulvany myograph system (620 M, Danish Myo Technology, Hinnerup, Denmark). Unstretched aortic rings were allowed to equilibrate for 30 min at 37 °C in 5 mL of KH. Next, the tension of the rings was increased stepwise to reach 10 mN and incubated in KH for another 20 min. After the normalization process was completed, the vessels’ contractile responses to potassium chloride (KCl 30 mM, 60 mM) and phenylephrine (Phe 3 × 10^−6^ M) were examined, to achieve the maximum contraction of each ring. The relaxation response of CO_G_ (60%) and CORM-A1 (500 µM) were assessed after 30 min in the presence of Phe. Control rings were similarly treated by adding equal doses of DMSO (control) to the organ bath. For each aorta ring, the endothelium-dependent response and endothelium-independent vasodilatation were assessed by adding cumulatively increasing concentrations of acetylcholine (ACh 10^−9^ to 10^−5^ M) or (SNP 10^−9^ to 10^−6^ M), respectively, precontracted with Phe. Vasodilator responses were expressed as a percentage of the previous tone induced by Phe in each case. All solutions were freshly prepared before the experiment. All reagents were prepared and diluted in distilled water.

### 4.8. Statistical Analysis

Statistical analysis was performed using ORIGINPRO 9.1 software (OriginLab Corporation, Northampton, MA, USA). The results are expressed as means +/−SD. Data were tested for normality. For statistical analysis one-way ANOVA was performed, with P values lower than 0.05 signed as *.

## Data Availability

Data sharing not applicable.

## Supplementary Materials

The following are available online at https://www.mdpi.com/article/10.3390/ijms22073584/s1.

## References

[1] True A.L., Olive M., Boehm M., San H., Westrick R.J., Raghavachari N., Xu X., Lynn E.G., Sack M.N., Munson P.J. (2007). Heme oxygenase-1 deficiency accelerates formation of arterial thrombosis through oxidative damage to the endothelium, which is rescued by inhaled carbon monoxide. Circ. Res..

[2] Tracz M.J., Juncos J.P., Grande J.P., Croatt A.J., Ackerman A.W., Katusic Z.S., Nath K.A. (2008). Induction of heme oxygenase-1 is a beneficial response in a murine model of venous thrombosis. Am. J. Pathol..

[3] Chen B., Guo L., Fan C., Bolisetty S., Joseph R., Wright M.M., Agarwal A., George J.F. (2009). Carbon monoxide rescues heme oxygenase-1-deficient mice from arterial thrombosis in allogeneic aortic transplantation. Am. J. Pathol..

[4] Tamura T., Kondo T., Ogawa K., Fukunaga K., Ohkohchi N. (2013). Protective effect of heme oxygenase-1 on hepatic ischemia-reperfusion injury through inhibition of platelet adhesion to the sinusoids. J. Gastroenterol. Hepatol..

[5] Sato K., Balla J., Otterbein L., Smith R.N., Brouard S., Lin Y., Csizmadia E., Sevigny J., Robson S.C., Vercellotti G. (2001). Carbon Monoxide Generated by Heme Oxygenase-1 Suppresses the Rejection of Mouse-to-Rat Cardiac Transplants. J. Immunol..

[6] Kramkowski K., Leszczynska A., Mogielnicki A., Chlopicki S., Grochal E., Mann B., Brzoska T., Urano T., Motterlini R., Buczko W. (2012). Antithrombotic properties of water-soluble carbon monoxide-releasing molecules. Arterioscler. Thromb. Vasc. Biol..

[7] Soni H., Jain M., Mehta A.A. (2011). Investigation into the mechanism(s) of antithrombotic effects of carbon monoxide releasing molecule-3 (CORM-3). Thromb. Res..

[8] Brüne B., Ullrich V. (1987). Inhibition of platelet aggregation by carbon monoxide is mediated by activation of guanylate cyclase. Mol. Pharmacol..

[9] Chlopicki S., Olszanecki R., Marcinkiewicz E., Lomnicka M., Motterlini R. (2006). Carbon monoxide released by CORM-3 inhibits human platelets by a mechanism independent of soluble guanylate cyclase. Cardiovasc. Res..

[10] Chlopicki S., Łomnicka M., Fedorowicz A., Grochal E., Kramkowski K., Mogielnicki A., Buczko W., Motterlini R. (2012). Inhibition of platelet aggregation by carbon monoxide-releasing molecules (CO-RMs): Comparison with NO donors. Naunyn. Schmiedebergs. Arch. Pharmacol..

[11] Kaczara P., Sitek B., Przyborowski K., Kurpinska A., Kus K., Stojak M., Chlopicki S. (2020). Antiplatelet effect of carbon monoxide is mediated by NAD+and ATP depletion. Arterioscler. Thromb. Vasc. Biol..

[12] Motterlini R., Sawle P., Hammad J., Bains S., Alberto R., Foresti R., Green C.J. (2005). CORM-A1: A new pharmacologically active carbon monoxide-releasing molecule. FASEB J..

[13] Foresti R., Braud L., Motterlini R. (2018). Chapter 7: Signaling by CO: Molecular and Cellular Functions. RSC Metallobiology.

[14] Brüne B., Schmidt K.U., Ullrich V. (1990). Activation of soluble guanylate cyclase by carbon monoxide and inhibition by superoxide anion. Eur. J. Biochem..

[15] Li L., Hsu A., Moore P.K. (2009). Actions and interactions of nitric oxide, carbon monoxide and hydrogen sulphide in the cardiovascular system and in inflammation—a tale of three gases!. Pharmacol. Ther..

[16] Stone J.R., Marletta M.A. (1994). Soluble Guanylate Cyclase from Bovine Lung: Activation with Nitric Oxide and Carbon Monoxide and Spectral Characterization of the Ferrous and Ferric States. Biochemistry.

[17] Ma X., Sayed N., Beuve A., Van Den Akker F. (2007). NO and CO differentially activate soluble guanylyl cyclase via a heme pivot-bend mechanism. EMBO J..

[18] Petrova O. (2017). Regulation, Activation, and Deactivation of Soluble Guanylate Cyclase and NO-Sensors. Doctoral Dissertation.

[19] Pyriochou A., Papapetropoulos A. (2005). Soluble guanylyl cyclase: More secrets revealed. Cell. Signal..

[20] Friebe A., Müllershausen F., Smolenski A., Walter U., Schultz G., Koesling D. (1998). YC-1 potentiates nitric oxide- and carbon monoxide-induced cyclic GMP effects in human platelets. Mol. Pharmacol..

[21] Cooper C.E., Brown G.C. (2008). The inhibition of mitochondrial cytochrome oxidase by the gasescarbon monoxide, nitric oxide, hydrogen cyanide and hydrogensulfide: Chemical mechanism and physiological significance. J. Bioenerg. Biomembr..

[22] Ishigami I., Zatsepin N.A., Hikita M., Conrad C.E., Nelson G., Coe J.D., Basu S., Grant T.D., Seaberg M.H., Sierra R.G. (2017). Crystal structure of CO-bound cytochrome c oxidase determined by serial femtosecond X-ray crystallography at room temperature. Proc. Natl. Acad. Sci. USA.

[23] D’Amico G., Lam F., Hagen T., Moncada S. (2006). Inhibition of cellular respiration by endogenously produced carbon monoxide. J. Cell Sci..

[24] Stojak M., Kaczara P., Motterlini R., Chlopicki S. (2018). Modulation of cellular bioenergetics by CO-releasing molecules and NO-donors inhibits the interaction of cancer cells with human lung microvascular endothelial cells. Pharmacol. Res..

[25] Prieto L., Rossier J., Derszniak K., Dybas J., Oetterli R.M., Kottelat E., Chlopicki S., Zelder F., Zobi F. (2017). Modified biovectors for the tuneable activation of anti-platelet carbon monoxide release. Chem. Commun..

[26] Peng L., Mundada L., Stomel J.M., Liu J.J., Sun J., Yet S.F., Fay W.P. (2004). Induction of heme oxygenase-1 expression inhibits platelet-dependent thrombosis. Antioxidants Redox Signal..

[27] Alshehri A., Bourguignon M.-P., Clavreul N., Badier-Commander C., Gosgnach W., Simonet S., Vayssettes-Courchay C., Cordi A., Fabiani J.-N., Verbeuren T.J. (2013). Mechanisms of the vasorelaxing effects of CORM-3, a water-soluble carbon monoxide-releasing molecule: Interactions with eNOS. Naunyn. Schmiedebergs. Arch Pharmacol..

[28] Wang R., Wang Z., Wu L. (1997). Carbon monoxide-induced vasorelaxation and the underlying mechanisms. Br. J. Pharmacol..

[29] Jaggar J.H., Leffler C.W., Cheranov S.Y., Tcheranova D.E.S., Cheng X. (2002). Carbon monoxide dilates cerebral arterioles by enhancing the coupling of Ca2+ sparks to Ca2+-activated K+ channels. Circ. Res..

[30] Failli P., Vannacci A., Mannelli L.D.C., Motterlini R., Masini E. (2012). Relaxant effect of a water soluble carbon monoxide-releasing molecule (CORM-3) on spontaneously hypertensive rat aortas. Cardiovasc. Drugs Ther..

[31] Naik J.S., Walker B.R. (2001). Homogeneous segmental profile of carbon monoxide-mediated pulmonary vasodilation in rats. Am. J. Physiol. Lung Cell. Mol. Physiol..

[32] Kiss L., Deitch E.A., Szabó C. (2008). Hydrogen sulfide decreases adenosine triphosphate levels in aortic rings and leads to vasorelaxation via metabolic inhibition. Life Sci..

[33] Griffith T.M., Edwards D.H., Newby A.C., Lewis M.J., Henderson A.H. (1986). Production of endothelium derived relaxant factor is dependent on oxidative phosphorylation and extracellular calcium. Cardiovasc. Res..

[34] Weir C.J., Gibson I.F., Martin W. (1991). Effects of metabolic inhibitors on endothelium-dependent and endothelium-independent vasodilatation of rat and rabbit aorta. Br. J. Pharmacol..

[35] Rodman D.M., Mallet J., McMurtry I.F. (1991). Difference in effect of inhibitors of energy metabolism on endothelium-dependent relaxation of rat pulmonary artery and aorta. Am. J. Respir. Cell Mol. Biol..

[36] Rotko D., Bednarczyk P., Koprowski P., Kunz W.S., Szewczyk A., Kulawiak B. (2020). Heme is required for carbon monoxide activation of mitochondrial BKCa channel. Eur. J. Pharmacol..

[37] Von Burg R. (1999). Carbon monoxide. J. Appl. Toxicol..

[38] Fayad-Kobeissi S., Ratovonantenaina J., Dabiré H., Wilson J.L., Rodriguez A.M., Berdeaux A., Dubois-Randé J.L., Mann B.E., Motterlini R., Foresti R. (2016). Vascular and angiogenic activities of CORM-401, an oxidant-sensitive CO-releasing molecule. Biochem. Pharmacol..

[39] Hasegawa U., Van Der Vlies A.J., Simeoni E., Wandrey C., Hubbell J.A. (2010). Carbon monoxide-releasing micelles for immunotherapy. J. Am. Chem. Soc..

[40] Wojcik T., Buczek E., Majzner K., Kolodziejczyk A., Miszczyk J., Kaczara P., Kwiatek W., Baranska M., Szymonski M., Chlopicki S. (2015). Comparative endothelial profiling of doxorubicin and daunorubicin in cultured endothelial cells. Toxicol. Vitr..

[41] Fedorowicz A., Buczek E., Mateuszuk L., Czarnowska E., Sitek B., Jasztal A., Chmura-Skirlińska A., Dib M., Steven S., Daiber A. (2018). Comparison of pulmonary and systemic NO- And PGI2-dependent endothelial function in diabetic mice. Oxid. Med. Cell. Longev..

