# Supplementary material for: Distinct Pharmacological Properties of Gaseous CO and CO-Releasing Molecule in Human Platelets
Source: Int J Mol Sci. 2021 Mar 30;22(7):3584. doi: 10.3390/ijms22073584 (PMC8037872; doi:10.3390/ijms22073584)
Supplement: Supplementary file 1 [file ijms-22-03584-s001.pdf]

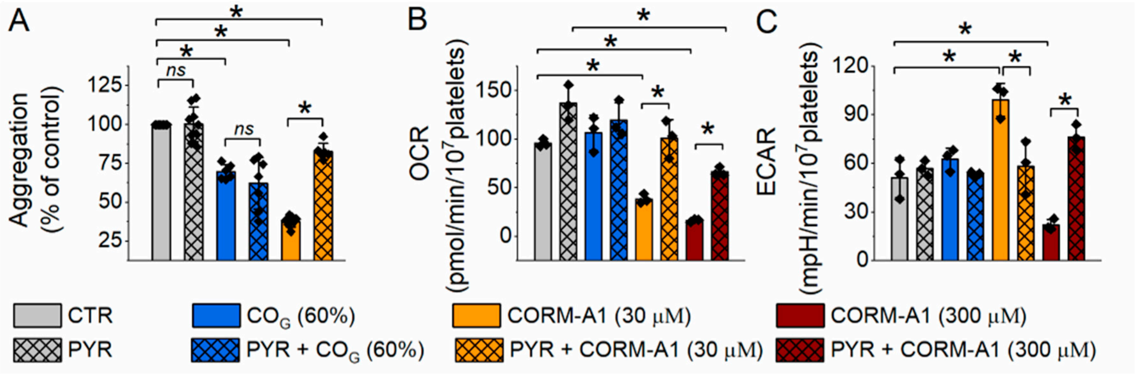


**Figure 1S**. Effects of pyruvate on CO–induced changes in platelet aggregation and energy metabolism. (A) Effects of Pyruvate (1 mM) on induced by collagen (2 µg/ml) aggregation of WP treated with $CO_G$ (60%) or CORM-A1 (30 µM). Data represent means +/-SD from et least three independent experiments. (B) Oxygen consumption rate (OCR) and (C) extracellular acidification rate (ECAR) were analyzed by Seahorse XF96 Analyzer. Platelets were untreated (CTR) or treated with pyruvate (1 mM) or $CO_G$ (60%) or CORM-A1 (30 or 300 µM) just before the start of the Seahorse assay; the readouts were taken 30 min after treatment. Data represent means +/-SD from et least three independent experiments. *$P < 0.05$.
